# Efficient achievement of enteral autonomy facilitates resolution of liver injury in necrotizing enterocolitis induced short bowel syndrome

**DOI:** 10.1038/s41598-022-22414-7

**Published:** 2022-10-20

**Authors:** Annika Mutanen, Ville Pöntinen, Riikka Gunnar, Laura Merras-Salmio, Mikko P. Pakarinen

**Affiliations:** 1 Department of Pediatric Surgery, Pediatric Liver and Gut Research Group, Pediatric Research Center, New Children’s Hospital, University of Helsinki and Helsinki University Hospital, Stenbäckinkatu 9, P.O Box 347, 00029 HUS Helsinki, Finland; 2 Department of Pediatric Gastroenterology, Pediatric Liver and Gut Research Group, Pediatric Research Center, New Children’s Hospital, University of Helsinki and Helsinki University Hospital, Helsinki, Finland

**Keywords:** Paediatric research, Gastrointestinal diseases, Hepatology

## Abstract

Children with short bowel syndrome (SBS) are at high risk for intestinal failure associated liver disease (IFALD). The aim of this retrospective follow-up study was to compare weaning off parenteral nutrition (PN) and IFALD between necrotizing enterocolitis (NEC) and non-NEC induced SBS. Altogether, 77 patients with neonatal SBS treated by our multidisciplinary intestinal failure unit (NEC n = 38, non-NEC SBS n = 39) were included and followed-up at least for 2 years until median age of 10 years (interquartile range, 6.0–16). Occurrence and characteristics of IFALD was assessed with liver biopsies obtained at median age of 3.2 (1.0–6.7) years (n = 62) and serum liver biochemistry. Overall, NEC patients had less end-jejunostomies and autologous intestinal reconstruction surgeries performed compared to non-NEC patients (< 0.05), while remaining small bowel anatomy was comparable between groups. Cumulative weaning off PN was more frequent and duration of PN shorter among NEC patients (P < 0.05). Overall cumulative probability of histological IFALD was lower among NEC patients during whole follow-up period (P = 0.052) and at 10 years (P = 0.024). NEC patients had lower ALT and GGT levels at last follow-up (P < 0.05 for all). In univariate Cox regression analysis, absence of end-jejunostomy, NEC diagnosis, longer remaining small bowel length, multidisciplinary treatment and prematurity were predictive for weaning off PN, while NEC diagnosis and lower birth weight in addition to multidisciplinary care protected from histological IFALD. Neonates with NEC induced SBS reached enteral autonomy earlier than those with non-NEC SBS, which associated with more efficient resolution of histological IFALD among long-term survivors.

## Introduction

In short bowel syndrome (SBS), extensive loss of functional gut mass leads to intestinal failure (IF) and long-term parenteral nutrition (PN) dependency^1^. Pediatric SBS is most often caused by necrotizing enterocolitis (NEC), mainly affecting premature and low birth weight infants, followed by midgut volvulus, gastroschisis, intestinal atresia and extended Hirschsprung’s disease^1^. Premature neonates with SBS, especially those with NEC, are at increased risk of cholestasis and intestinal failure associated liver disease (IFALD)^2,3^. In a recent study over 20% of surgical NEC patients with biochemical cholestasis died^4^, while significant histopathological IFALD is present in approximately 50% of pediatric SBS patients at median age of five years^5^.

Prematurity not only impairs liver function, but also its ability to respond to many risk factors of IFALD a neonate with SBS is exposed to^6^. These risk factors include prolonged PN, high rates of central line and intestinal sepsis episodes, lack of enteral feeds and disruption of enterohepatic circulation^1,5^. The immature liver has reduced canalicular transport and bile acid detoxification capacity which together with increased susceptibility to lipid peroxidation and systemic infections predisposes premature infants to liver injury^6–8^. Intestinal inflammation, impaired intestinal barrier function, bacterial translocation and impaired portal blood flow may further increase the risk of liver disease in NEC patients^9^. On the other hand, early achievement of enteral autonomy may protect NEC patients from progressive IFALD^10^.

Long-term liver outcomes in NEC induced neonatal SBS are yet to be studied. The aim of this retrospective comparative follow-up study was to assess long-term biochemical and histopathological liver outcomes in pediatric SBS due to NEC. We hypothesized, that due to vulnerability of immature liver, SBS caused by NEC is associated with slower recovery of histopathological IFALD when compared to other etiologies of pediatric SBS.

## Methods

### Ethics

This study has an ethical approval by the Helsinki University Hospital (Helsinki, Finland) ethics committee. All experiments were performed in accordance with relevant guidelines and regulations. An informed consent was obtained from all patients and/or their legal guardian(s).

### Patients and study design

All patients treated in the Children’s Hospital, Helsinki University Hospital for pediatric IF were reviewed, and SBS patients born between years 1988 to 2018 were identified. SBS was defined as PN requirement for over 60 consecutive days and/or surgical removal > 50% of age adjusted small intestine^11^. All patients had undergone a bowel resection and non-operatively treated NEC patients were not included in the study. Overall, 78 SBS patients were identified, of whom 77 with available follow-up data were included (Table 1). Of them, 62 patients had a liver biopsy obtained. Patients with NEC as the underlying etiology (NEC-SBS n = 38) were compared to those with other causes of SBS (non-NEC-SBS n = 39). The other causes of SBS included midgut volvulus (n = 13), small bowel atresia (n = 9), extended Hirschsprung’s disease (n = 8), gastroschisis with atresia (n = 6) and isolated gastroschisis (n = 3).

**Table 1:** Patient characteristics and liver biochemistry.

	All SBS patients	NEC-SBS	NON-NEC-SBS	p-value
Patients, n	77	38	39	
Follow-up age, y	10.0 (6.0–15.6)	8.0 (6.0–10.3)	14.0 (8.0–17.0)	**0.023**
Gestational age, wk	34 (26–37)	26 (25–31)	37 (35–39)	** < 0.001**
Birth weight, kg	1.6 (0.76–3.2)	0.76 (0.63–1.1)	3.2 (2.3–3.6)	** < 0.001**
Small bowel length, cm	40 (25–60)	40 (25–58)	40 (25–78)	0.596
Small bowel length, %	25 (17–43)	29 (17–48)	23 (16–35)	0.175
Serum citrulline, umol/L*	17 (11–26)	17 (10–26)	17 (11–25)	0.816
ICV preserved, n	37 (48)	18 (47)	19 (49)	1.000
Colon remaining, %	90 (57–100)	94 (60–100)	90 (50–100)	0.482
Intestinal circuit, J/JC/JIC	10/28/39	1/18/19	9/10/20	**0.013**
AIR surgery n (%)	23 (30)	6 (16)	17 (44)	**0.012**
Intestinal transplantation, n (%)	2 (0)	0 (0)	2 (0)	0.497
Survival, n (%)	72 (94)	36 (95)	36 (92)	1.000
Age at PN start, days	0 (0)	0 (0)	0 (0)	0.059
Current PN, n (%)	19 (25)	6 (16)	13 (33)	0.112
Duration of PN, mo	11.7 (5.0–37.8)	8.2 (4.6–23.3)	19.4 (7.4–79.4)	**0.022**
Septic episodes, n	1 (0–2)	1 (0–2)	0 (0–2)	0.198
Start of IF treatment before 2009, n (%)**	31 (40)	8 (21)	23 (61)	**0.001**
ALT, U/L	30 (19–50)	22 (19–42)	37 (23–66)	**0.029**
Above UNL, n (%)	22 (29)	14 (37)	8 (21)	0.135
AST, U/L	43 (33–62)	42 (36–61)	43 (32–73)	0.941
Above UNL, n (%)	19 (25)	11 (29)	8 (21)	0.785
GTT, U/L	16 (11–29)	12 (10–22)	17 (11–47)	**0.025**
Above UNL, n (%)	12 (16)	5 (13)	7 (18)	0.754
Bilirubin, umol/L	8 (5–12)	8 (5–11)	7 (5–16)	0.654
Above UNL, n (%)	9 (12)	5 (13)	4 (10)	0.736
Bilirubin, conjugated, umol/L	3 (2–5)	3 (2–5)	4 (2–7)	0.245
Above UNL, n (%)	11 (14)	6 (16)	5 (13)	0.754
APRI	0.37 (0.25–0.61)	0.36 (0.30–0.57)	0.40 (0.24–0.87)	0.692

Significant values are in bold.

The data are recorded at last follow-up visit or before intestinal transplantation (n = 2) or death (n = 5). Values are medians (interquartile range) or frequencies. P-values are Mann Whitney U-test or Fisher’s exact test.

*AIR* Autologous intestinal reconstruction, *ALT* alanine aminotransferase, *APRI* AST-to-platelet ration index, *AST* aspartate aminotransferase, *GTT* gamma-glutamyl transferase, *ICV* Ileocecal valve, *J* end-jejunostomy, *JC* jejunocolic anastomosis, *JIC* jejunoileocolic anastomosis, *NEC* necrotizing enterocolitis, *PN* parenteral nutrition, *SBS* short bowel syndrome, *UNL* upper normal limit.

*First available measurement (n = 67) at median age 1.0 years (IQR 0.3–5.8). ** A standardized multidisciplinary management and follow-up program was implemented in 2009.

Data were prospectively collected from 2010 onwards and retrospectively before 2010 (last follow up date was before year 2010 in 4 patients). Since 2009, a standardized multidisciplinary management and follow-up program including modern fish oil containing lipid emulsions, antimicrobial catheter locks, autologous reconstructive (AIR) surgery (serial transverse enteroplasty, STEP, and longitudinal intestinal lengthening and tailoring, LILT), and intestinal transplantation has been running in our center^11,12^. As described in detail previously, liver biopsies are routinely used to assess IFALD within our IF rehabilitation program^5^.

Clinical data, including gestational age and weight, anatomy of the remaining bowel, surgical procedures and PN duration (from start to weaning off) were collected from the patient records. Number of blood culture positive septic episodes was recorded from birth to follow-up. Percentage age-adjusted small bowel and colon length was calculated based on published age-specific normal values^13,14^. Follow-up continued until the end of 2020 allowing for at least two years follow up for each patient after end of study inclusion period in 2018. The follow up data was collected at the latest follow up visit (n = 70) or at last follow up before intestinal transplantation (n = 2) or death (n = 5).

### Serum biochemistry

Blood samples were drawn after overnight fast. Alanine aminotransferase (ALT), aspartate aminotransferase (AST), gamma glutamyl transferase (GGT), total and conjugated bilirubin, platelet count, and bile acids were analyzed using standard hospital laboratory methods. The APRI index was calculated [(AST/upper limit of normal) × 100 /platelet count (10^9^/L)]^15^. Serum citrulline, a marker of bowel enterocyte mass, was measured by using an automatic amino acid analyzer (Biochromon 30 Physiological and Midas Autosampler, Biochromon Limited, Cambridge, England) as described earlier^16^.

### Liver biopsies and histopathology

Core needle liver biopsies were taken under general anesthesia with ultrasound guidance for diagnosis or follow-up during surveillance intestinal endoscopies or planned laparotomies^5,17^. As described previously, follow-up liver biopsies were obtained when previous biopsy showed abnormal and potentially progressive histopathology^5^. Liver biopsies were analyzed by two experienced pediatric liver pathologists to consensus according to a standardized histopathological protocol^5,17^. As liver biopsies contained median 15 (5–22) portal areas they were considered representative. The biopsies were scored for cholestasis (grade 0 to 3; absent, minimal, marked, prominent), portal inflammation (grade 0 to 4; absent, minimal, mild, moderate, prominent), steatosis (grade 0 to 3; < 25%, 25–50%, > 50% of hepatocytes affected) and fibrosis (Metavir stage from 0 to 4) as previously described^5^. IFALD was defined as any abnormal finding in liver biopsy, including cholestasis, portal inflammation, fibrosis or steatosis. Active IFALD was defined as presence of histological cholestasis and/or portal inflammation and chronic IFALD as presence of Metavir fibrosis stage ≥ 2 and/or steatosis grade ≥ 2 without cholestasis or inflammation^5^.

### Statistical analyses

IBM SPSS statistics, version 25 was used for data analysis. All results are presented as medians with interquartile range (IQR), unless stated otherwise. Mann–Whitney U-test and Fisher’s exact test was used to compare statistical significances between the two groups. Kaplan–Meier log rank survival analysis was used to evaluate cumulative occurrence of IFALD and weaning off PN. Predictive factors for weaning off PN and the presence of histopathological IFALD were analyzed with univariate and multivariate Cox regression analyses. Statistical significance is defined as p-value < 0.05.

## Results

### Patient characteristics and survival

Patient characteristics are shown in Table 1. As expected, NEC patients had significantly lower gestational age and birth weight. Overall, 23 patients underwent AIR surgery, significantly more frequently in non-NEC group, and two non-NEC patients with extended Hirschsprung’s disease received intestinal transplantation. Overall survival was 94% and comparable between groups (Table 1). Cause of death was sepsis in three patients (1 NEC, 2 non-NEC) and IFALD in two non-NEC patients both occurring before year 2008. The number of blood culture positive septic episodes from birth to follow-up was not significantly different between groups, while a higher proportion non-NEC patients started their treatment before 2009 (Table 1).

### Anatomy of remaining bowel

As shown in Table 1, the proportion of patients with end-jejunostomy was significantly higher in non-NEC group (23% vs. 2.6%, P = 0.014) at the expense of jejuno-colic anastomoses (26% vs. 47%, P = 0.060), while the proportion of jejuno-ileo-colic anastomoses were comparable (51% vs. 50%, P = 1.000). The remaining absolute and age-adjusted small bowel length, proportion of remaining colon, presence of ileocecal valve and the first available serum citrulline levels were not significantly different between groups.

### PN dependency

**Figure 1:**
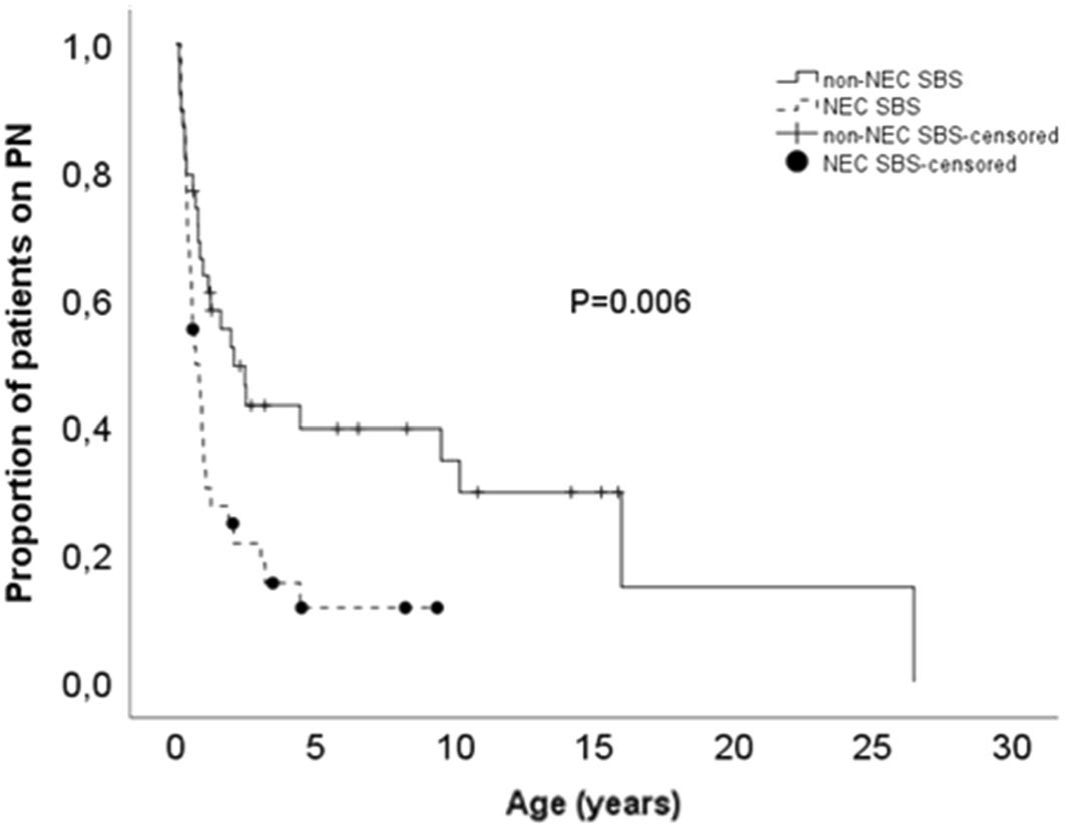
Cumulative proportion of PN dependent patients in NEC (n = 38) and non-NEC (n = 39) group.

Patients with NEC weaned off PN more effectively when compared to non-NEC patients, as shown in Kaplan Meier analysis in Fig. 1. After the minimum follow-up of 2 years 74% (95%CI 0.747–1.186) of NEC patients and 49% (95%CI 1.120–1.591) of non-NEC patients had weaned off PN (P = 0.021). The respective figures at 5 years and 10 years after PN start were 84% (95%CI 0.280–1.112) versus 59% (95%CI 0.352–3.789; P = 0.009) and 84% (95%CI 0.280–1.112) versus 67% (95%CI 0.352–3.785; P = 0.012), respectively. Median time to weaning off PN was 0.7 (95%CI 0.3–1.1) years in NEC compared to 2.1 years (95%CI 0.9–3.3) in non-NEC-SBS (P = 0.006).

In univariate Cox regression analysis for weaning off PN at 5 years after PN start, diagnosis of NEC, longer remaining age-adjusted small bowel length, absence of end-jejunostomy, start of IF treatment after implementation of the standardized multidisciplinary management and follow-up program in 2009 and lower gestational age were all predictive for weaning off PN (Table 2). In multivariate Cox regression model, presence of end-jejunostomy and shorter small bowel length remained significant risk factors for prolonged PN dependency (Table 2).

**Table 2:** Cox regression analysis for weaning of PN at five years.

	Univariate model				Multivariate model			
Covariate	B	Hazard ratio	95% CI	P-value	B	Hazard ratio	95% CI	P-value
NEC diagnosis	0.790	2.204	1.182–4.112	**0.013**	− 0.510	0.601	0.261–1.381	0.230
Small bowel%	0.042	1.043	1.027–1.058	** < 0.001**	0.046	1.047	1.032–1.062	** < 0.001**
End-jejunostomy	− 2.359	0.094	0.013–0.689	**0.020**	− 2.084	0.124	0.028–0.549	**0.006**
Number of septic episodes	− 0.079	0.924	0.818–1.044	0.203				
Start of treatment before 2009	0.651	1.918	1.097–3.351	**0.022**	− 0.010	0.990	0.522–1.880	0.997
Gestational age	− 0.070	0.933	0.885–0.982	**0.009**	0.043	1.044	0.976–1.116	0.212
Birth weight	0.000	1.000	0.999–1.000	**0.001**				

Significant values are in bold.

*NEC* necrotizing enterocolitis, *PN* parenteral nutrition, *small bowel %* age-adjusted small bowel length.

### Liver disease

All together 62 patients had a liver biopsy obtained (Table 3). Median age at liver biopsy was 3.2 (1.0–5.2) years in NEC group and 3.7 (0.8–9.6) years in non-NEC group (P = 0.578). Any histopathological signs of IFALD, defined by presence of cholestasis, portal inflammation, fibrosis or steatosis in liver biopsy, was found in 65% of NEC and 84% of non-NEC patients (P = 0.146) (Table 3). Although occurrence and grade of portal inflammation, fibrosis and steatosis were higher among non-NEC patients the differences were not statistically significant (Table 3).

**Table 3:** Liver histopathological findings.

	NEC-SBS	NON-NEC-SBS	P-value
Abnormal liver biopsy, n (%)	20 (65)	26 (84)	0.146
Active IFALD, (%)	9 (29)	12 (39)	0.592
Chronic IFALD, (%)	8 (26)	9 (29)	1.000
Cholestasis, n (%)	8 (26)	8 (26)	1.000
Grade, mean (range)	0.4 (0–3)	0.5 (0–3)	0.847
Portal inflammation, n (%)	5 (16)	9 (29)	0.363
Grade, mean (range)	0.3 (0–3)	0.7 (0–3)	0.146
Fibrosis, n (%)	16 (52)	21 (68)	0.300
Metavir stage, mean (range)	1.0 (0–4)	1.2 (0–4)	0.318
Steatosis, n (%)	9 (29)	14 (45)	0.293
Grade, mean (range)	0.3 (0–2)	0.8 (0–3)	0.076

Values are medians (interquartile range) or frequencies unless otherwise indicated. P-values are for Mann Whitney U test or Fisher’s exact test.

*IFALD* intestinal failure associated liver disease, *NEC* necrotizing enterocolitis, *SBS* short bowel syndrome.

**Figure 2:**
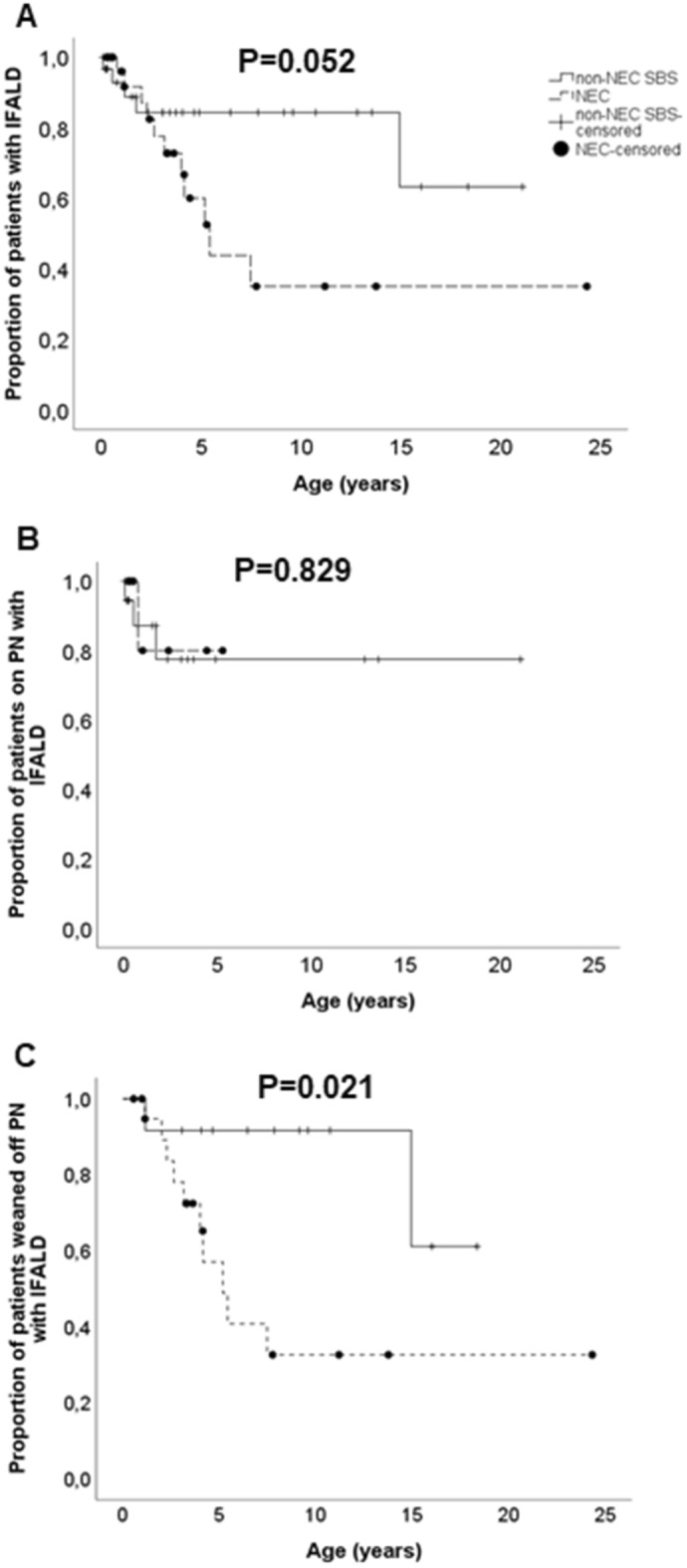
Cumulative proportion of patients with histological IFALD in NEC (n = 31) and non-NEC (n = 31) group. (**A**) All patients, (**B**) Patients currently on PN, (**C**) Patients who had weaned off PN.

Histological IFALD resolved earlier among NEC patients as they showed lower cumulative occurrence of histological IFALD during follow-up compared to non-NEC SBS patients (Fig. 2). At 10 years the proportion of NEC and non-NEC patients with histological IFALD was 37% (95%CI 4.259–7.384) vs 87% (7.297–9.865, P = 0.024). As the occurrence of histological IFALD was comparable between the groups among PN-dependent patients, the faster normalization of liver histology was achieved by earlier weaning off PN in NEC group (Fig. 2). In univariate Cox regression analysis including current PN dependency, duration of PN, gestational age, birth weight, remaining age-adjusted small bowel length, presence of end-jejunostomy, start of IF treatment before implementation of the standardized multidisciplinary management and follow-up program in 2009 and underlying diagnosis as variables, non-NEC diagnosis, higher birth weight and start of IF treatment before 2009 predicted presence of histological IFALD at 10 years (Table 4). In multivariate model, only the start of treatment before 2009 remained significant (Table 4).

**Table 4:** Cox regression analysis for presence of histological IFALD at 10 years.

	Univariate model				Multivariate model			
Covariate	B	Hazard ratio	95% CI	P-value	B	Hazard ratio	95% CI	P-value
Non-NEC SBS	1.161	3.194	1.010–10.098	**0.048**	0.564	0.689	0.111–2.7814	0.689
Weaned off PN	− 1.421	0.242	0.032–1.842	0.170				
Duration of PN	− 0.001	0.999	0.999–1.000	0.074				
Small bowel%	0.015	1.015	0.990–1.041	0.243				
No end-jejunostomy	− 3.238	0.039	0.000–24.243	0.323				
Start of treatment before 2009	3.478	32.410	3.906–268.949	**0.001**	3.452	31.573	2.472–403.293	**0.008**
Gestational age	− 0.081	0.992	0.835–1.019	0.111				
Birth weight	− 0.001	0.999	0.999–1.000	**0.046**	0.000	0.678	0.999–1.001	0.678

Significant values are in bold.

*NEC* necrotizing enterocolitis, *SBS* short bowel syndrome, *PN* parenteral nutrition, *small bowel %* age-adjusted small bowel length.

Liver biochemistry values were determined at end of follow-up (Table 1). Although transaminase levels were increased in 25–29%, GGT in 16% and bilirubin in 12% of patients, their median values were within normal range in both groups (Table 1). Median ALT and GGT levels were significantly higher in non-NEC patients in relation to NEC patients (Table 1). ALT [62 (33–145) vs. 31 (21–44), P = 0.005], AST [51 (37–237) vs. 40 (33–48), P = 0.010), and GGT [52 (29–101) vs. 15 (11–46), P = 0.001] were higher in PN dependent patients compared to patients weaned off PN.

## Discussion

To our best knowledge, this is one of the first studies to assess long-term occurrence of histopathological IFALD in relation to regaining of enteral autonomy in neonatal SBS. Our results demonstrate that neonates with NEC induced SBS wean off PN earlier compared to neonates with SBS due to other underlying etiologies. Efficient regaining of enteral autonomy in NEC induced SBS associated with faster resolution of histological liver injury and lower levels of ALT and GGT at the end of follow-up.

In accordance with our findings, NEC has been previously shown to predict earlier achievement of enteral autonomy in children with SBS^18,19^. In our study, 74% and 84% of NEC-SBS patients weaned off PN by 2 and 5 years as opposed to 49% and 59% among non-NEC patients. Sparks et al. reported that 65% of NEC versus 29% of non-NEC SBS children reached enteral autonomy after median follow up of 4.3 years^10^. In contrast to other underlying diagnoses of SBS, patients with NEC also continued to wean off PN even after 36 months at similar rate as during the first 12 months^10,18^.

In the current study, NEC diagnosis, absence of end-jejunostomy, longer remaining small intestine, multidisciplinary treatment after 2009, and prematurity (lower gestational age and weight) independently predicted weaning off PN. When compared to other patients with SBS, patients with NEC were more frequently premature and had less often end-jenunostomy, while serum citrulline concentrations and other key features of intestinal anatomy such small intestinal length, proportion of remaining colon and presence of ICV were comparable between groups. Collectively, these findings indicate that prematurity and almost uniform establishment of intestinal continuity essentially contributed to earlier achievement of enteral autonomy in NEC patients over others. Less AIR procedures and intestinal transplantations were also performed among NEC patients, indicating better functional status of the remaining bowel among them. Premature babies with SBS are thought to possess increased capacity for adaptation due to greater growth potential of the remaining intestine compared to their full term counterparts^13^. This facilitates enteral tolerance and absorption, promoting earlier achievement of enteral autonomy^18,20,21^. The importance of re-establishment of intestinal continuity for reaching enteral autonomy is well established^1,22^. In SBS patients with double enterostomy chyme reinfusion into remaining ileum and colon promotes mucosal health, improves nutrient absorption and shortens duration of PN while relieving cholestasis and normalizing bile acid metabolism^22–24^.

Our main new finding, against our initial hypothesis, was that resolution of histological IFALD occurred earlier and associated with lower serum ALT and GGT levels at the latest follow-up in patients with NEC induced SBS. Increased serum GGT levels associate with the presence of cholestasis and/or inflammation in the liver^5^. In Cox regression analysis, NEC etiology of SBS, lower birth weight and multidisciplinary treatment after 2009 predicted resolution of histological IFALD during follow-up. Even though NEC patients have an increased risk of cholestasis and biochemical liver dysfunction at early stages of SBS^2,3,25^, earlier discontinuation of PN allowed faster recovery of liver injury among them, whereas during PN delivery histological IFALD occurred at similar frequency in both groups. Our findings confirm accomplishment of enteral autonomy as an effective way to prevent and manage IFALD^20,25^. While around 30% of our patients showed increased serum liver biochemistry at the end of follow-up, most commonly ALT values, much smaller proportion normalized liver histology, which consisted of cholestasis, portal inflammation, fibrosis or steatosis comparably in both groups. Accordingly, in a previous study of children with SBS serum ALT levels remained elevated in over one third of patients including those who had weaned off PN after a median follow-up of 3.8 years^26^. These findings highlight the need for further studies to unravel the significance and pathogenesis of IFALD following weaning off PN.

Benefits of multidisciplinary IF care have been recognized previously^12,27^. Accordingly, implementation of our multidisciplinary IF rehabilitation program since 2009 associated with improved results for reaching enteral autonomy and resolution of IFALD in neonates with SBS. From this retrospective study, it is challenging to identify which specific components of our redefined management protocol mostly contributed to these improvements in outcomes, but introduction of fish-oil containing parenteral lipids, efficient prevention of sepsis episodes along with dedicated multidisciplinary approach might have been among the most important factors^27–30^. Here, treatment of non-NEC SBS patients started more frequently before year 2009, which may have negatively affected long-term outcomes of these patients in relation to NEC patients. Our study was also limited by its retrospective design and relatively small number of patients. Although the wide inclusion period may have modified our results, liver biopsies were obtained at similar ages in both groups and great majority of them after standardization of the treatment in 2009. Due to lack of paired liver biopsies, we were not able to address longitudinal changes in individual patients but had to rely on survival curve estimations of resolution of histological IFALD.

In conclusion, our study indicates that neonates with NEC induced SBS reach enteral autonomy earlier than those with non-NEC SBS. The earlier achievement of enteral autonomy and standardized multidisciplinary care associated with earlier resolution of histological IFALD.

## Supplementary Information

Supplementary Figure 1.

## Data Availability

The data is available on request from Dr. Annika Mutanen, annika.mutanen@hus.fi, Department of Pediatric Surgery, Pediatric Liver and Gut Research Group, Pediatric Research Center, New Children's Hospital, University of Helsinki and Helsinki University Hospital, Stenbackinkatu 9, PO Box 347, 00029 HUS, Helsinki, Finland.

## Abbreviations

AIR: Autologous reconstructive (AIR) surgery
ALT: Alanine aminotransferase
AST: Aspartate aminotransferase
GGT: Gamma glutamyl transferase
IF: Intestinal failure
IFALD: Intestinal failure associated liver disease
LILT: Longitudinal intestinal lengthening and tailoring
NEC: Necrotizing enterocolitis
SBS: Short bowel syndrome
STEP: Serial transverse enteroplasty
