# Supplementary figures and images for: Efficient achievement of enteral autonomy facilitates resolution of liver injury in necrotizing enterocolitis induced short bowel syndrome
Source: Sci Rep. 2022 Oct 20;12:17516. doi: 10.1038/s41598-022-22414-7 (PMC9584958; doi:10.1038/s41598-022-22414-7)

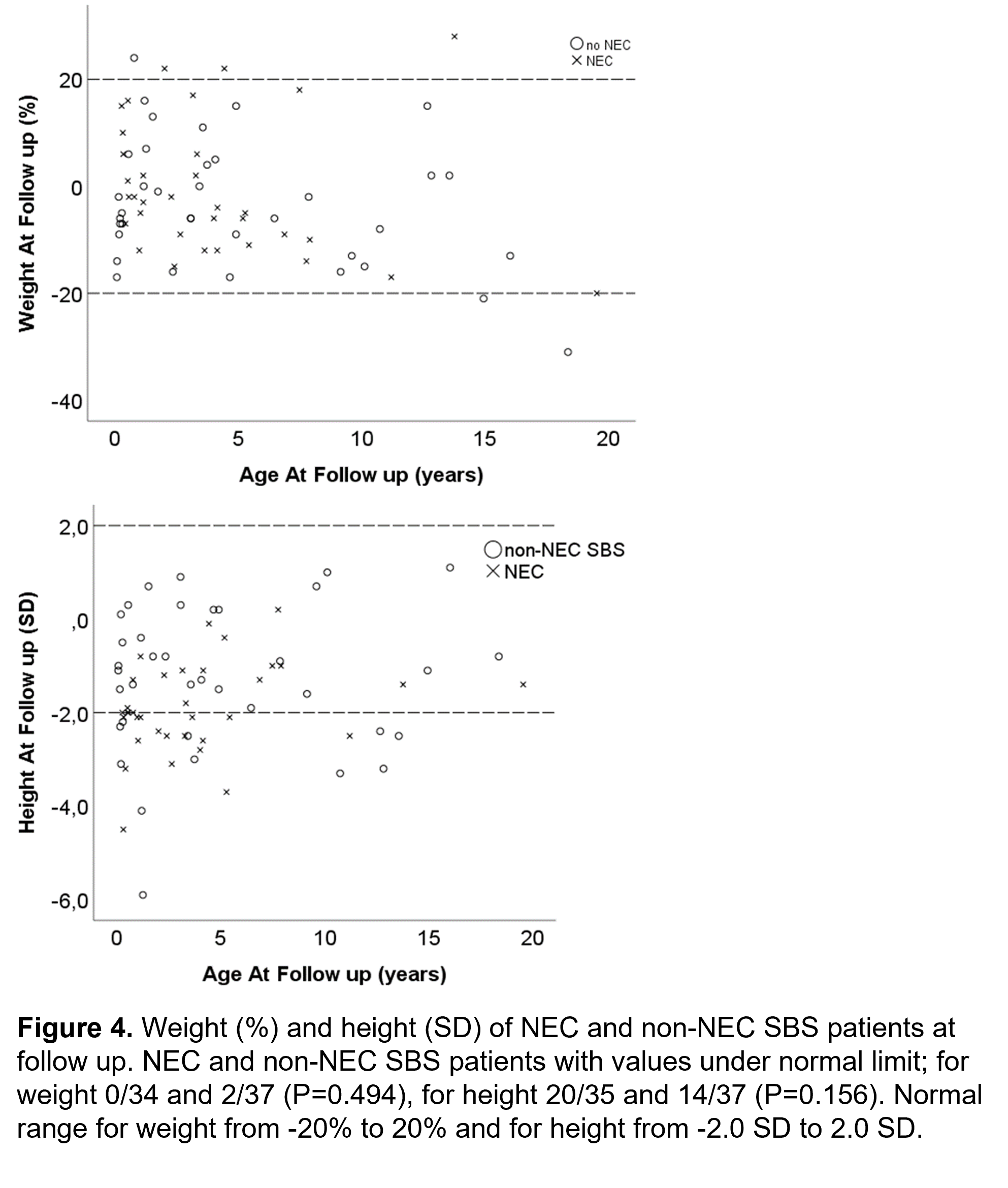

Supplement: Supplementary file 1 — Supplementary Figure 1. [file 41598_2022_22414_MOESM1_ESM.tif]
